# Real-world identification of high-risk medications for drug-induced osteoporosis: a multi-method pharmacovigilance study based on FAERS with WHO Vigibase validation

**DOI:** 10.3389/fendo.2026.1783284

**Published:** 2026-04-13

**Authors:** Wenbin Gao, Erqing Tan, Jian Zhu, Wei Liu, Jiping Sun, Zhongyi Su

**Affiliations:** 1 Department of Internal Medicine, Shanxi Bethune Hospital, Shanxi Academy of Medical Sciences, Third Hospital of Shanxi Medical University, Tongji Shanxi Hospital, Taiyuan, China; 2 Department of Orthopedics, Shanxi Bethune Hospital, Shanxi Academy of Medical Sciences, Third Hospital of Shanxi Medical University, Tongji Shanxi Hospital, Taiyuan, China

**Keywords:** adverse event onset, drug-induced osteoporosis, FAERS, high-risk drugs, LASSO regression, pharmacovigilance

## Abstract

**Objective:**

Using the FAERS database in conjunction with the WHO Vigibase, this study systematically evaluated the characteristics, high-risk drugs, and demographic factors associated with drug-induced osteoporosis, and analyzed the onset time patterns of adverse events for major implicated medications.

**Methods:**

Osteoporosis-related reports from FAERS between Q1–2014 and Q1–2025 were collected, and after deduplication, 55,895 cases were included. Disproportionality analyses (ROR, PRR, MGPS, BCPNN) and LASSO regression were applied to identify high-risk drugs. Multivariate regression was used to assess the independent effects of age, sex, and drug exposure. Onset time distribution analyses were conducted for the five drugs with the strongest signals, and external validation was performed using WHO Vigibase.

**Results:**

Reports of osteoporosis increased markedly after 2020, with females and individuals aged ≥60 years representing high-risk populations. Disproportionality analysis identified 18 associated drugs, with Tenofovir Disoproxil showing the highest number of reports and the strongest signal (ROR 367.06). LASSO regression further identified 17 strongly associated drugs, predominantly antineoplastic agents and immunosuppressants. Multivariate analysis indicated that age >60 years (OR = 3.7) and female sex (OR = 1.8) were significant risk factors, while Tenofovir Disoproxil (OR = 210) and Adefovir (OR = 59) exhibited the strongest drug-related risk signals. Onset time analysis revealed distinct temporal patterns among the five drugs: Tenofovir Disoproxil and Medroxyprogesterone had the highest cumulative risk and the broadest time distribution, whereas Adefovir and Anastrozole were mainly associated with early-onset events. External validation in WHO Vigibase corroborated the high-risk signals of Tenofovir Disoproxil and Adefovir.

**Conclusions:**

This comprehensive pharmacovigilance study identifies key medications associated with elevated osteoporosis risk and reveals marked differences in onset time patterns. These findings provide robust evidence to support targeted monitoring and inform individualized preventive strategies in clinical practice.

## 1 Introduction

Osteoporosis is a metabolic bone disorder characterized by decreased bone mineral density and deterioration of bone microarchitecture, leading to reduced bone strength and a markedly increased risk of fractures (1). With the acceleration of global population aging, the prevalence of osteoporosis continues to rise, representing a major public health concern that adversely affects the health and quality of life of older adults. According to the World Health Organization (WHO), over 200 million people worldwide are estimated to be affected by osteoporosis, with incidence increasing significantly with age (2, 3). Women, particularly postmenopausal women, constitute a high-risk population for this disease (4, 5).

The etiology of osteoporosis involves a combination of endogenous and exogenous factors, including genetic background, hormonal changes, inadequate nutritional intake, physical inactivity or sedentary lifestyle, smoking, and excessive alcohol consumption (6, 7). In recent years, with the widespread use of pharmacological treatments for chronic diseases, drug-induced osteoporosis (DOP) has received increasing attention. DOP is characterized by abnormal reductions in bone mineral density and an elevated risk of fractures, and it may occur even in patients without a prior history of osteoporosis. Multiple drug classes have been implicated in the development of osteoporosis, including glucocorticoids, antiepileptic agents, proton pump inhibitors, certain chemotherapeutic drugs, and immunosuppressants (8–10). However, DOP is often underestimated in clinical practice due to its long latency period and atypical or subtle clinical manifestations. Despite its clinical significance, existing studies have largely focused on the mechanisms of individual drugs or risk assessments based on limited clinical cases, lacking a comprehensive overview of the overall drug spectrum and quantification of risk intensity. Large-scale, real-world studies systematically elucidating the associations between medications and osteoporosis remain scarce. This limitation significantly impedes a full understanding of DOP and constrains evidence-based guidance for rational drug use in clinical practice.

The present study aims to systematically identify drug-related risk signals associated with osteoporosis using the FDA Adverse Event Reporting System (FAERS) database through disproportionality analyses. High-risk drugs were further screened using least absolute shrinkage and selection operator (LASSO) regression, and their risk characteristics were evaluated via multivariate regression and adverse event onset time distribution analyses. This study provides insights into the potential risk spectrum of DOP, offering evidence to support safer clinical prescribing, optimize individualized treatment strategies, and ultimately reduce the risk of adverse outcomes.

## 2 Materials and methods

### 2.1 Data source and collection

Data from the first quarter of 2014 to the first quarter of 2025 were obtained from the FAERS. Each quarterly release consists of seven datasets: DEMO (demographic and administrative information), REAC (MedDRA-coded adverse events), DRUG (reported drugs and biological products), OUTC (patient outcomes), RPSR (report sources), THER (drug therapy start and end dates), and INDI (MedDRA-coded indications). Duplicate reports were removed in accordance with FDA recommendations using CASEID, FDA_DT, and PRIMARYID. For records with the same CASEID, the report with the most recent FDA_DT was retained; if both CASEID and FDA_DT were identical, the record with the larger PRIMARYID was kept. A secondary deduplication was performed to remove residual duplicate PRIMARYIDs.

Adverse events were identified using MedDRA preferred terms (PTs). Osteoporosis-related PTs included osteoporosis, bone density decreased, osteopenia, osteoporotic fracture, postmenopausal osteoporosis, senile osteoporosis, and bone density abnormal. Drugs were categorized according to their role in the reports (primary suspected drug, secondary suspected drug, concomitant drug, or interacting drug). After excluding medications indicated for the treatment of osteoporosis, only reports in which the drug was designated as the primary suspected drug were included in the subsequent analysis. Statistical significance was evaluated using Fisher’s exact test with Bonferroni correction. Volcano plots were generated with the logarithm of the odds ratio (log(ROR)) on the x-axis and the negative logarithm (base 10) of the adjusted p value [−log10(p-adjusted)] on the y-axis.

### 2.2 Drug screening

FAERS reports containing complete patient demographic information (age and sex) were extracted, and only cases with non-missing data were included in the analysis. Reports with patient age >80 years were considered outliers and excluded. Univariate analyses were first performed for all suspected drugs. Drugs with a p value <0.01 were preliminarily selected and subsequently entered into LASSO regression for variable selection. The drugs retained by the LASSO model, together with basic patient characteristics, were then included as independent variables in a multivariate logistic regression model to identify risk factors associated with osteoporosis-related adverse drug reactions.

For the drugs identified through screening in the FAERS database, the number of adverse event reports related to osteoporosis was further manually extracted from the WHO VigiBase database, and the corresponding reporting odds ratios were calculated to complement the findings derived from the FAERS-based analysis. The WHO VigiBase database compiles spontaneous adverse event reports from more than 180 countries and is widely used for global pharmacovigilance signal detection. Although VigiBase does not provide access to detailed case-level information or allow filtering analyses for specific countries, its extensive geographic coverage offers a highly heterogeneous global dataset that facilitates cross-database consistency assessments. Therefore, the external validation in this study primarily focused on comparing the consistency of signal strength and reporting trends across different databases. This approach helps mitigate potential biases associated with reliance on a single database and further enhances the robustness of the study findings.

### 2.3 Statistical analysis

Four disproportionality analysis methods were employed in this study to identify potential drug–event association signals, including the reporting odds ratio (ROR), the proportional reporting ratio (PRR), the Bayesian confidence propagation neural network (BCPNN), and the multi-item gamma Poisson shrinker (MGPS). These methods are all based on a 2 × 2 contingency table, in which the target drug–event pairs are compared with all other drug–event combinations to detect potential positive signals (**Table 1**). The detailed calculation formulas for each method are presented in **Table 2**. While frequency-based methods are straightforward and computationally efficient, Bayesian approaches are more computationally demanding but have demonstrated superior performance in minimizing false-positive signals, particularly in the setting of sparse adverse event reporting. The combined use of these complementary algorithms was intended to improve the robustness and credibility of signal identification. All data management and statistical analyses were conducted using R software (version 4.4.1).

**Table 1:** Four-grid table of disproportionality analysis method.

Type of drug	Target adverse events	All other adverse events	Total
Target drugs	a	b	a+b
All other drugs	c	d	c+d
Total	a+c	b+d	a+b+c+d

a: number of reports of the target adverse event associated with the target drugs;

b: number of reports of all other adverse events associated with the target drugs;

c: number of reports of the target adverse event associated with all other drugs;

d: number of reports of all other adverse events associated with all other drugs.

**Table 2:** Principle of disproportionality analysis and standard of signal detection.

Algorithms	Equation	Criteria
ROR	$ROR\;=\;\frac{ad}{bc}$	95%CI>1, a≥3
	$95\%CI=\;e^{\ln\;(ROR)\pm \sqrt[{1.96}]{\left(\frac{1}{a}+\frac{1}{b}+\frac{1}{c}+\frac{1}{d}\right)}}$	
PRR	$PRR\;=\;\frac{a/(a+b)}{c/(c+d)}$	PRR≥2, χ ^2^≥4, a≥3
	$\chi^{2}\;=\;\frac{\left[{\left(ad-bc\right)}^{2}](a+b+c+d\right)}{\left(a+b)(c+d)(a+c)(b+d\right)}$	
MGPS	$EBGM\;=\;\frac{a(a+b+c+d)}{\left(a+c)(a+b\right)}$	EBGM05>2, a>0
	$EBGM\;=\;e^{\ln\;(EBGM)-\sqrt[{2}]{1.64(\frac{1}{a}+\frac{1}{b}+\frac{1}{c}+\frac{1}{d})}}$	
BCPNN	$IC\;=\;\log_{2}\;\frac{a(a+b+c+d)}{\left(a+c)(a+b\right)}$	IC025>0
	$IC_{025}\;=\;e^{\ln\;(IC)-1.96\;\sqrt{\left(\frac{1}{a}+\frac{1}{b}+\frac{1}{c}+\frac{1}{d}\right)}}$	

## 3 Results

### 3.1 Descriptive analysis

**Figure 1:**
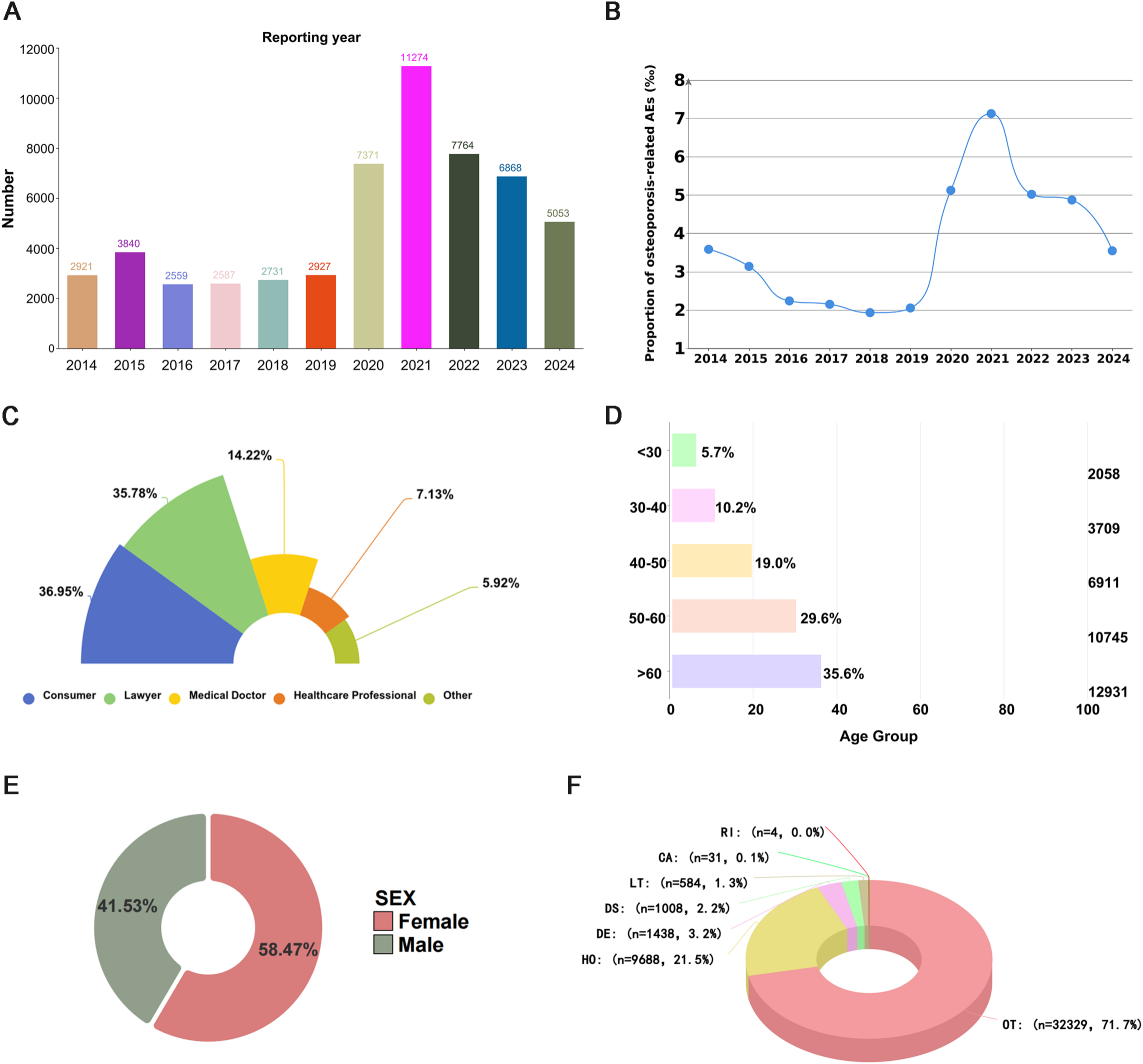
Overall reporting characteristics of drug-associated osteoporosis adverse events in the FAERS database. **(A)** Distribution of adverse event reports by year. **(B)** Proportion (‰) of osteoporosis-related AEs among total FAERS reports. **(C)** Distribution of adverse event reports by reporter occupation. **(D)** Distribution of adverse event reports by age group. **(E)** Distribution of adverse event reports by sex. **(F)** Distribution of adverse event reports by clinical outcome.

A total of 55,895 osteoporosis-related adverse drug reaction reports, involving 919 primary suspect drugs, were included after deduplication, covering the period from Q1–2014 to Q1 2025. To explore potential associations between patient characteristics and osteoporosis, reports were stratified by year, sex, age, reporting source, and outcomes. Before 2020, the number of osteoporosis-related adverse events remained relatively stable, followed by a sharp increase starting in 2020, with the highest number reported in 2021 (11,274 cases) (**Figure 1A**). To account for the increasing total number of FAERS reports over time, we further calculated the yearly proportion of osteoporosis-related adverse events. The results showed a similar increasing trend after 2020, with a peak in 2021, supporting the robustness of the observed trend (**Figure 1B**). The majority of reports originated from the United States (73.00%), followed by Canada (8.80%). Most reports were submitted by consumers (36.95%) and lawyers (35.78%)(**Figure 1C**).Analysis by age revealed that adverse event reporting increased with age, with patients aged ≥60 years (12,931 cases, 35.57%) representing a high-risk group for osteoporosis (**Figure 1D**). Regarding sex distribution, after excluding reports with unknown sex, females accounted for 58.5% of cases, exceeding males (41.5%), which may reflect the higher incidence of postmenopausal osteoporosis in women (**Figure 1E**). Among all reports, the most frequently observed outcomes were other serious outcome(OT) and Hospitalization (HO) (**Figure 1F**). Detailed data are available in **Supplementary Material Table 1**.

### 3.2 Disproportionality analysis of drugs strongly associated with osteoporosis

**Figure 2:**
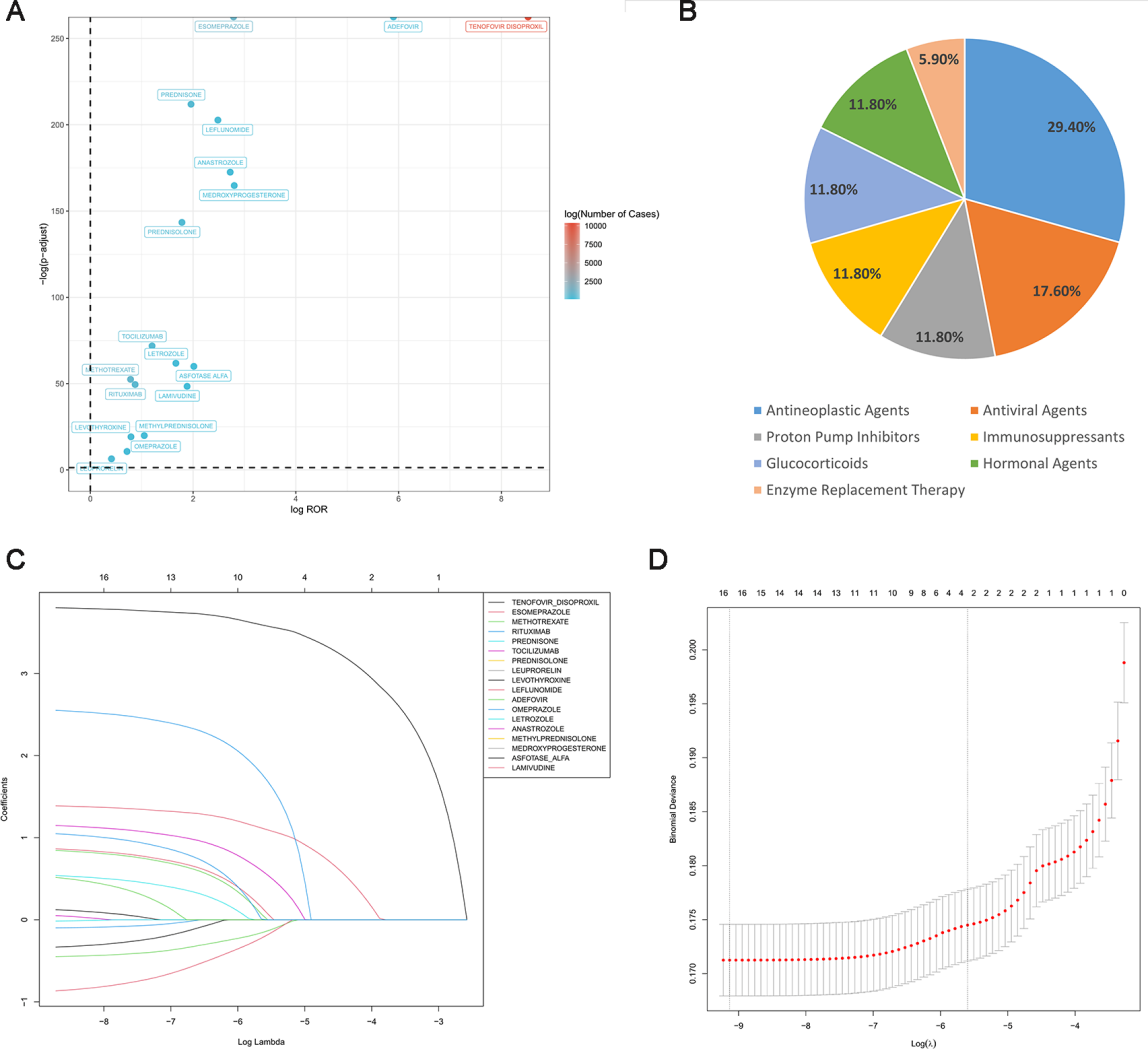
Drug screening and classification results for osteoporosis-related adverse reactions. **(A)** Volcano plot of drug screening. **(B)** Distribution of drug classifications. **(C, D)** Feature selection process based on the LASSO regression model.

To more accurately identify drugs potentially inducing osteoporosis, disproportionality analyses were performed, resulting in the identification of 18 drugs associated with osteoporosis. Among these, Tenofovir Disoproxil had the highest number of reported cases (10,349) and the strongest signal (ROR 367.06, 95% CI 356.32–378.12), followed by Adefovir, Medroxyprogesterone, Esomeprazole, and Anastrozole. The screening results are illustrated in a volcano plot (**Figure 2A**). These drugs spanned multiple therapeutic categories, with antineoplastic agents representing the largest proportion (29.4%), followed by immunosuppressants (17.6%) (**Figure 2B**).

**Figure 3:**
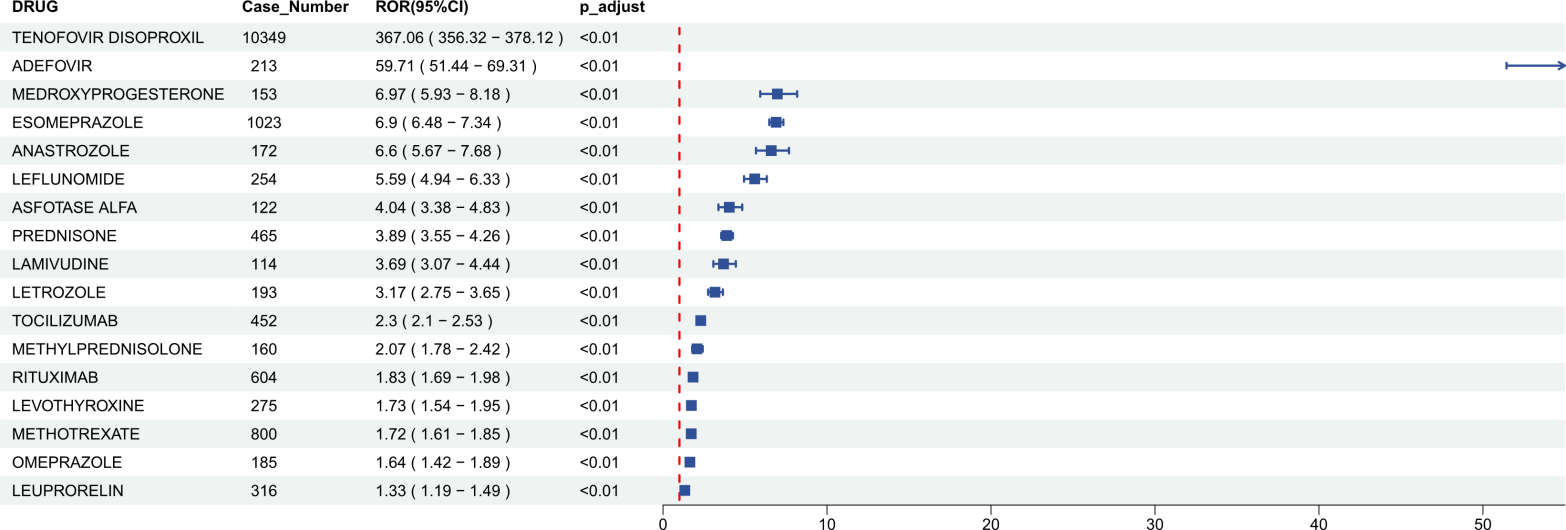
Forest plot of odds ratios for drugs associated with osteoporosis.

Subsequently, the LASSO machine learning algorithm was applied, further identifying 17 drugs considered strongly associated with osteoporosis-related adverse events (**Figures 2C, D**). Among them, Tenofovir Disoproxil exhibited the strongest signal, and a forest plot illustrates the signal strength for each drug (**Figure 3**). Notably, upon reviewing the respective drug labels, only six drugs explicitly mention the risk of osteoporosis. Six drugs (Tenofovir Disoproxil, Adefovir, Esomeprazole, Leuprorelin, Levothyroxine, and Omeprazole) did not explicitly list “osteoporosis” but indirectly referenced a potential risk. The remaining five drugs (Asfotase Alfa, Lamivudine, Leflunomide, Rituximab, and Tocilizumab) did not report osteoporosis or bone mineral density reduction as adverse events.

### 3.3 Multivariate regression analysis

Multivariate regression analysis indicated that, after adjusting for confounding factors, increasing age and female sex were significant demographic risk factors for osteoporosis. Specifically, individuals aged >60 years (OR = 3.7, 95% CI: 3.3–4.1) and those aged 50–60 years (OR = 3.0, 95% CI: 2.7–3.4) exhibited the highest risk, while females had a 1.8-fold higher risk compared to males (95% CI: 1.7–1.9).

**Figure 4:**
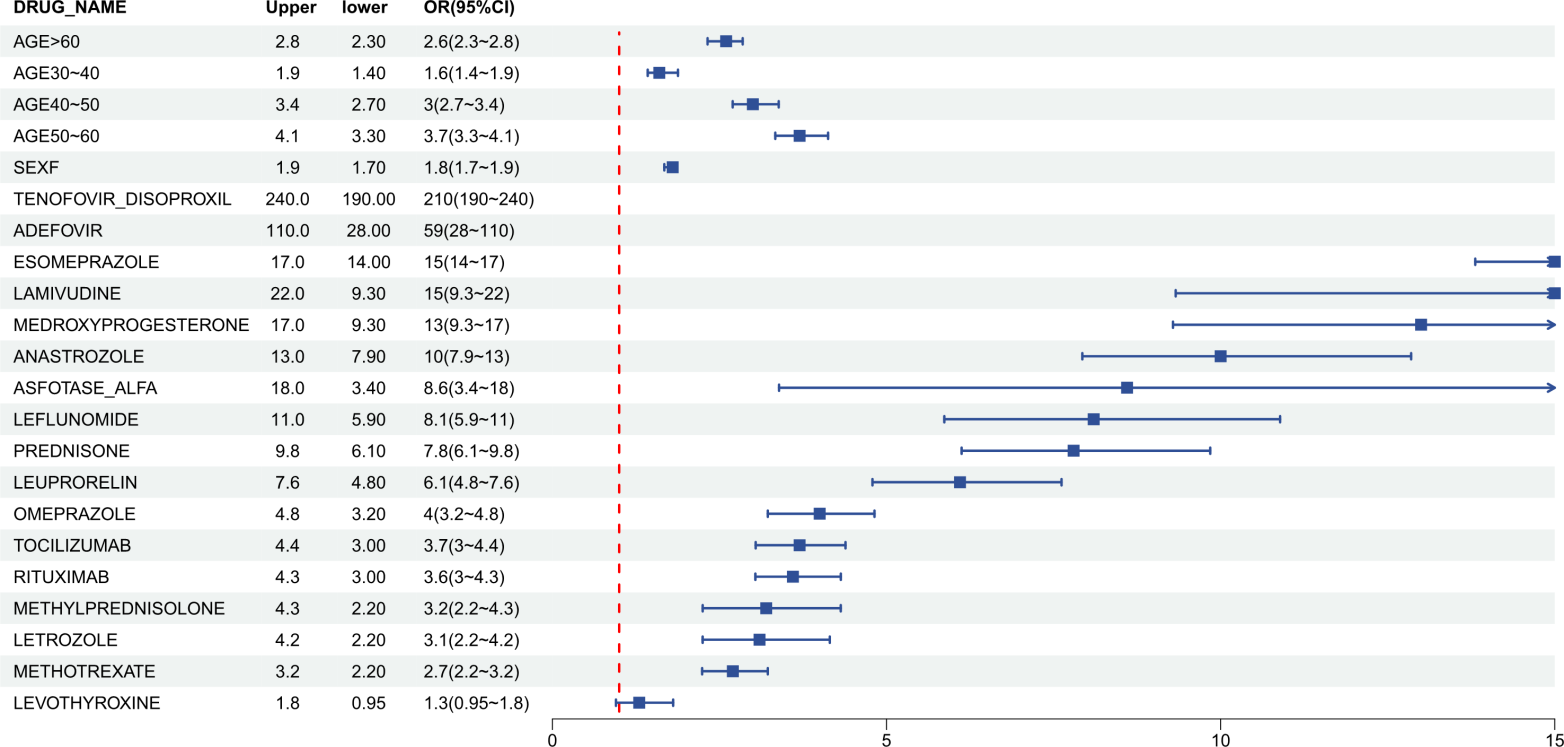
Results of the multivariable logistic regression analysis.

Regarding medications, after adjusting for age, sex, and other factors, several drugs demonstrated strong risk signals. Tenofovir Disoproxil exhibited an exceptionally high association with osteoporosis (OR = 210, 95% CI: 190–240), followed by Adefovir (OR = 59, 95% CI: 28–110). Additionally, Esomeprazole (OR = 15), Lamivudine (OR = 15), Medroxyprogesterone (OR = 13), and Anastrozole (OR = 10) all had adjusted OR values ≥10, indicating a very strong association with osteoporosis risk (**Figure 4**).

### 3.4 Onset time of drug-induced osteoporosis

**Figure 5:**
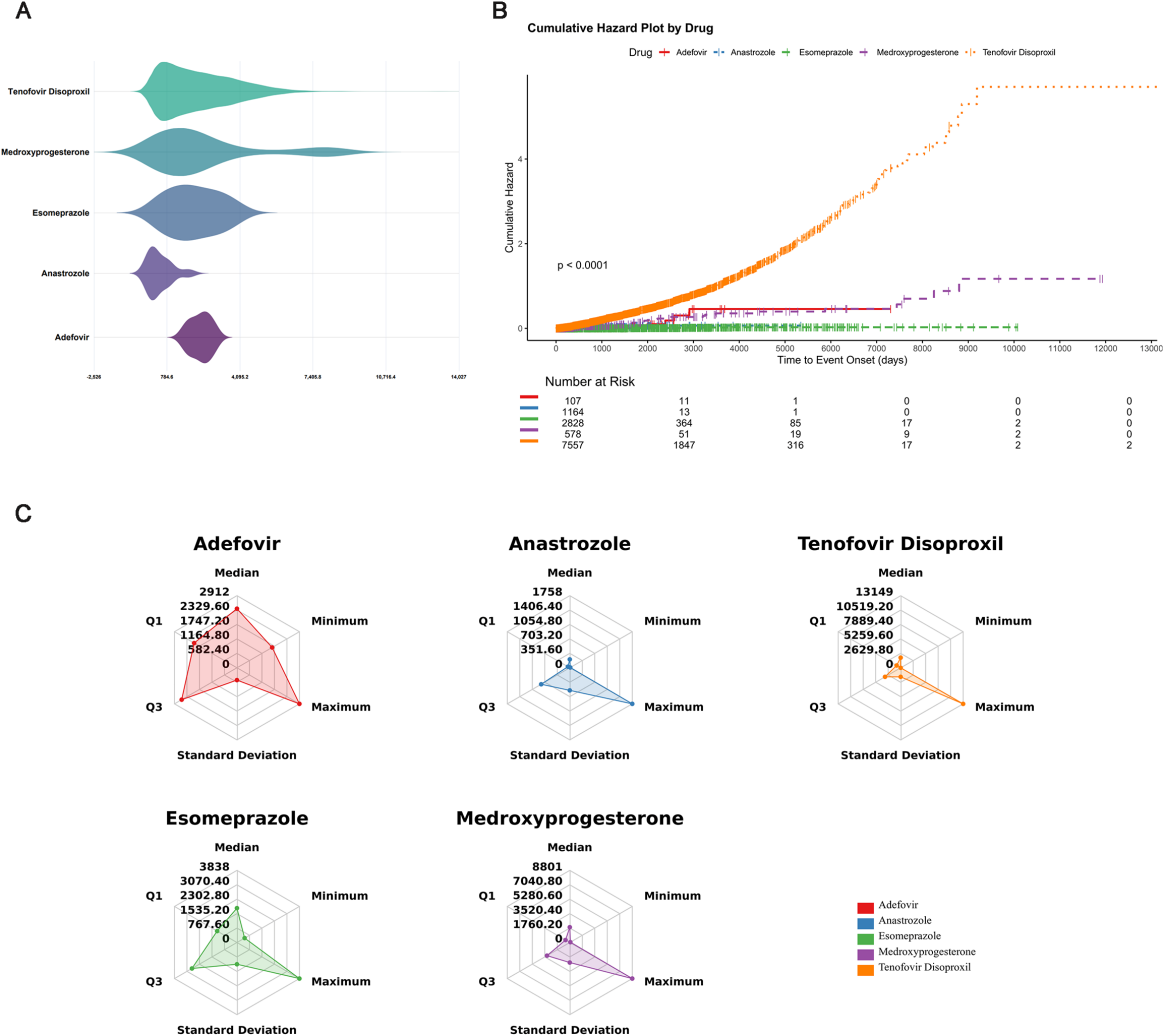
Temporal characteristics of drug-induced osteoporosis adverse events. **(A)** Violin plots. **(B)** Cumulative risk curves. **(C)** Radar chart illustrating temporal patterns.

We further evaluated the onset time characteristics of osteoporosis-related adverse events for the five drugs with the strongest signals: Tenofovir Disoproxil, Adefovir, Medroxyprogesterone, Esomeprazole, and Anastrozole. The results revealed significant heterogeneity in the timing of adverse events among these drugs, as illustrated by the violin plots (**Figure 5A**). **Figure 5B** shows that the cumulative incidence was highest in the Tenofovir Disoproxil group, with a rapid increase during the early treatment period and continued risk accumulation over time, indicating a strong association with osteoporosis development. The Medroxyprogesterone group exhibited the second-highest cumulative risk, showing a gradual upward trend, also suggesting substantial risk. In contrast, the cumulative incidence for Esomeprazole, Adefovir, and Anastrozole was relatively low, indicating a comparatively lower risk. Overall, Tenofovir Disoproxil and Medroxyprogesterone appear to be major contributors to drug-induced osteoporosis, underscoring the need for enhanced clinical monitoring and risk management.

The radar plot visualized the temporal distribution of osteoporosis events induced by the five drugs (**Figure 5C**). The analysis revealed distinct temporal risk patterns among different medications. Tenofovir Disoproxil exhibited the largest radar area, with the highest median and standard deviation, indicating that events tended to occur later and with considerable interindividual variability. Medroxyprogesterone also showed a dispersed distribution, suggesting potential delayed or cumulative effects. In contrast, Adefovir and Anastrozole displayed relatively concentrated onset times, predominantly occurring in the early treatment period, warranting close monitoring during initial therapy. The temporal characteristics of Esomeprazole were intermediate, without a pronounced early or delayed trend. Overall, osteoporosis events associated with Tenofovir Disoproxil and Medroxyprogesterone tended to occur later and were widely distributed, whereas Adefovir and Anastrozole events were mostly early-onset and concentrated.

### 3.5 External validation using the WHO Vigibase database

To minimize bias arising from reliance on a single data source, this study further conducted external validation using the WHO Vigibase database, covering the period from 2000 to 2024. The results showed that Tenofovir Disoproxil and Adefovir consistently exhibited strong signals across multiple disproportionality metrics, including ROR, PRR, MGPS, and BCPNN (**Table 3**), which was highly consistent with the FAERS-based findings and further supports their robust association with osteoporosis. However, Esomeprazole and Anastrozole displayed relatively weak signals in Vigibase and did not reproduce the high-intensity associations observed in FAERS.

**Table 3:** External validation of osteoporosis-related drug signals in the WHO Vigibase database.

Drug	PT	ROR(95%Cl)	PRR(χ²)	EBGM(EBGM05)	BCPNN
Tenofovir Disoproxil	Osteoporosis	**6.3 (6.03 - 6.58)**	**6.18 (8972.26)**	**3.06 (2.95)**	**1.61 (1.56)**
	Osteopenia	**9.82 (9.09 - 10.61)**	**9.73 (5112.64)**	**3.58 (3.35)**	**1.84 (1.76)**
	Osteoporotic fracture	**4.81 (3.22 - 7.19)**	**4.81 (71.98)**	2.75 (1.96)	**1.46 (0.97)**
	Bone density decreased	**57.18 (53.16 - 61.5)**	**54.5 (38601.75)**	**4.72 (4.44)**	**2.24 (2.2)**
Adefovir	Osteoporosis	**3.1 (2.99 - 3.21)**	**3.05 (4478.66)**	**2.28 (2.21)**	**1.19 (1.14)**
	Osteopenia	**3.57 (3.38 - 3.78)**	**3.55 (2377.07)**	**2.49 (2.38)**	**1.32 (1.25)**
	Osteoporotic fracture	**2.75 (1.96 - 3.86)**	**2.75 (37.5)**	**2.13 (1.61)**	**1.09 (0.63)**
	Bone density decreased	**4.87 (4.74 - 5)**	**4.68 (15961.78)**	**2.91 (2.84)**	**1.54 (1.51)**
Medroxyprogesterone	Osteoporosis	0.33 (0.3 - 0.36)	0.33 (628.47)	0.37 (0.35)	-1.42 (-1.55)
	Osteopenia	0.46 (0.4 - 0.52)	0.46 (144.78)	0.5 (0.45)	-1 (-1.19)
	Osteoporotic fracture	**1.8 (1.13 - 2.86)**	1.8 (6.39)	1.6 (1.09)	**0.68 (0.03)**
	Bone density decreased	0.07 (0.06 - 0.08)	0.07 (1792)	0.08 (0.07)	-3.69 (-3.94)
Esomeprazole	Osteoporosis	0.36 (0.34 - 0.38)	0.36 (2011.39)	0.55 (0.53)	-0.86 (-0.93)
	Osteopenia	0.16 (0.15 - 0.18)	0.17 (1777.76)	0.3 (0.28)	-1.74 (-1.88)
	Osteoporotic fracture	0.15 (0.08 - 0.26)	0.15 (58.52)	0.27 (0.17)	-1.88 (-2.68)
	Bone density decreased	0.04 (0.04 - 0.04)	0.04 (10993.35)	0.08 (0.08)	-3.58 (-3.72)
Anastrozole	Osteoporosis	0.34 (0.31 - 0.38)	0.34 (395.72)	0.37 (0.34)	-1.44 (-1.61)
	Osteopenia	0.52 (0.44 - 0.6)	0.52 (74.79)	0.54 (0.48)	-0.88 (-1.1)
	Osteoporotic fracture	0.48 (0.19 - 1.18)	0.48 (2.68)	0.51 (0.24)	-0.98 (-2.2)
	Bone density decreased	0.1 (0.09 - 0.12)	0.1 (1019.94)	0.12 (0.1)	-3.12 (-3.37)

Bold font indicates positive signals.

## 4 Discussion

Osteoporosis is a common metabolic bone disorder characterized by an imbalance between bone formation and bone resorption during the process of bone remodeling (11, 12). Estrogen deficiency represents the most important endocrine driver and promotes osteoclastogenesis by regulating the receptor activator of nuclear factor-κB ligand (RANKL)/osteoprotegerin (OPG) axis and enhancing the activity of inflammatory cytokines, while simultaneously suppressing osteoblast function, thereby accelerating bone loss (13–17). Nutritional and metabolic status also play a critical role. Vitamin D deficiency and insufficient calcium and phosphate intake can impair bone mineralization and induce secondary hyperparathyroidism, while chronic kidney disease, metabolic acidosis, and hypophosphatemia may further exacerbate osteoporosis (18–20). In addition, age-related decline in bone formation capacity and various endocrine abnormalities may further aggravate disturbances in bone metabolism (21–23). In recent years, drug exposure has been increasingly recognized as an important exogenous factor, as certain medications can induce drug-induced osteoporosis by interfering with the bone remodeling process (8, 24). Against this background, the present study systematically evaluated adverse event reports associated with osteoporosis from 2014 to 2024 using the FAERS database, with the aim of providing evidence to inform the clinical management of DOP.

The results showed that reports of osteoporosis-related adverse events have increased steadily since 2020. This trend may be associated with several factors during the COVID-19 pandemic, including increased use of certain medications, reduced physical activity, delays in the management of chronic diseases, and heightened awareness of pharmacovigilance. From a geographical perspective, most reports originated from North America and Europe, which is consistent with the more established adverse drug reaction surveillance systems in these regions. In terms of demographic characteristics, individuals aged ≥65 years and females accounted for the majority of reports, which is in line with the epidemiological profile of osteoporosis. This finding suggests that aging and postmenopausal estrogen deficiency may represent important susceptibility backgrounds for drug-related disturbances in bone metabolism. Notably, most reports were submitted by patients and legal representatives rather than healthcare professionals, suggesting that osteoporosis, as an insidious and slowly progressive adverse event, may be underestimated in routine clinical practice. This observation highlights the importance of strengthening proactive pharmacovigilance and drug safety monitoring.

At the drug level, Tenofovir Disoproxil showed the most prominent signal, demonstrating the highest risk strength across all analytical indicators. Tenofovir Disoproxil is a nucleoside reverse transcriptase inhibitor (NRTI) widely used for the antiviral treatment of HIV-1 infection and chronic hepatitis B virus infection (25, 26). Previous studies have shown that Tenofovir Disoproxil can accumulate in renal tubular cells and induce mitochondrial dysfunction, leading to impaired phosphate reabsorption, chronic hypophosphatemia, and defective bone mineralization (27–29). In addition, it may inhibit osteoblast differentiation while enhancing osteoclast activity, thereby accelerating bone loss (30). Similarly, prolonged exposure to Adefovir may cause proximal renal tubular toxicity, leading to hypophosphatemia and impaired synthesis of active vitamin D, which may subsequently contribute to bone loss and an increased risk of osteoporosis (31–33). It should be noted that patients receiving Tenofovir Disoproxil or Adefovir therapy are typically affected by HIV infection or chronic hepatitis B (34, 35), conditions that themselves may increase the risk of osteoporosis through mechanisms such as chronic inflammation, metabolic disturbances, and altered nutritional status. Therefore, some of the observed drug–osteoporosis associations in real-world studies may be influenced by underlying diseases, a phenomenon commonly referred to as indication bias or confounding by indication. Careful consideration of these potential confounders is essential when interpreting the results. In addition, several immunomodulatory agents, including Methotrexate, Rituximab, and Tocilizumab, also exhibited signals in this study. These agents may interfere with bone metabolism by affecting the RANKL/OPG axis and inflammatory pathways, or through concomitant use with glucocorticoids that enhance bone resorption (36–39). Time-to-onset analysis further provided important information for clinical risk surveillance. The results indicated that adverse events associated with Tenofovir Disoproxil tended to occur relatively early and showed a gradually increasing cumulative probability, suggesting that bone metabolic changes should be monitored even during short-term therapy. In contrast, Medroxyprogesterone demonstrated a more pronounced cumulative effect, indicating that long-term monitoring may be more appropriate. The differences in time-to-onset among these drugs suggest that drug-related bone metabolic injury may follow distinct temporal patterns, which may help inform individualized monitoring strategies. In Vigibase, Tenofovir Disoproxil and Adefovir showed significant high-risk signals, confirming their strong association with osteoporosis. In contrast, Esomeprazole and Anastrozole had weaker signals and did not replicate the high-risk signals seen in FAERS. This difference likely reflects systematic variations between pharmacovigilance databases rather than lower true risk. Future studies should combine real-world data with prospective cohorts, mechanistic experiments, and clinical trials to further validate the safety of these drugs.

It is noteworthy that, among the 17 drugs identified in this study as being significantly associated with osteoporosis, five drugs do not explicitly list osteoporosis or decreased bone mineral density as reported adverse reactions in their prescribing information, suggesting that these associations may represent potential unexpected signals. At present, the mechanisms by which these drugs may influence bone metabolism remain complex and have not been fully elucidated. Asfotase alfa, an enzyme replacement therapy used for the treatment of hypophosphatasia, exerts its therapeutic effect by restoring tissue-nonspecific alkaline phosphatase activity and improving bone mineralization (40, 41). Given the critical role of alkaline phosphatase in skeletal mineralization, alterations in its activity may theoretically influence the bone remodeling process. However, clear evidence regarding the impact of this drug on the risk of osteoporosis remains limited, and whether it exerts broader skeletal effects warrants further investigation. Lamivudine is generally considered to have a favorable skeletal safety profile (42). Skeletal changes observed in populations receiving long-term antiviral therapy may more likely reflect the combined effects of underlying diseases and overall antiviral treatment rather than the direct effect of Lamivudine itself. In addition, prolonged antiviral therapy may affect renal tubular function and phosphate reabsorption, thereby indirectly interfering with bone metabolism. Consequently, the potential impact of Lamivudine on osteoporosis risk remains uncertain. The immunosuppressive agent Leflunomide exerts its effects by inhibiting the key enzyme dihydroorotate dehydrogenase in the *de novo* pyrimidine synthesis pathway, thereby suppressing the proliferation of activated lymphocytes and modulating immune responses (43). Since pro-inflammatory cytokines such as TNF-α and IL-6 play key roles in osteoclast differentiation and bone resorption, alterations in these inflammatory pathways may theoretically affect the dynamic balance between osteoblast and osteoclast activity (44). Similarly, the biologic agents Rituximab and Tocilizumab target B-cell and IL-6 signaling pathways, respectively, and these immune pathways interact with the RANKL/OPG axis, a key regulatory system involved in bone remodeling (45–47). Although these mechanisms suggest potential links between immune modulation and bone metabolism, clinical evidence demonstrating a direct effect of these drugs on osteoporosis risk remains limited. It should also be noted that, in real-world clinical practice, patients often receive multiple medications simultaneously, and some commonly co-administered drugs, such as glucocorticoids, proton pump inhibitors, or antiepileptic agents, may themselves increase the risk of osteoporosis. However, in spontaneous reporting databases such as the FDA Adverse Event Reporting System, information on concomitant medications is often incomplete, making it difficult to fully control for these potential confounding factors. Therefore, the drug-related signals identified in this study should primarily be interpreted as pharmacovigilance signals rather than evidence of a confirmed causal relationship.

Despite providing systematic evidence based on large real-world pharmacovigilance databases, this study has several inherent limitations. First, as spontaneous reporting systems, the FAERS and VigiBase databases are susceptible to reporting bias, including underreporting, selective reporting, and potential duplicate records. Second, these databases lack important clinical variables, such as drug dosage, treatment duration, baseline bone mineral density, concomitant medications, and patient adherence, all of which may influence bone metabolism and limit causal inference. In addition, some of the observed drug–osteoporosis associations may be confounded by underlying diseases or treatment indications. Future prospective cohort studies, clinical trials, and mechanistic investigations are needed to further validate these associations and to clarify their potential biological mechanisms.

## Data Availability

The original contributions presented in the study are included in the article/**Supplementary Material**. Further inquiries can be directed to the corresponding author.
